# Neutrophil-to-lymphocyte ratio as a prognostic biomarker for patients with locally advanced esophageal squamous cell carcinoma treated with definitive chemoradiotherapy

**DOI:** 10.1038/srep42581

**Published:** 2017-02-14

**Authors:** Xi-Lei Zhou, Yong-Qiang Li, Wei-Guo Zhu, Chang-Hua Yu, Ya-Qi Song, Wan-Wei Wang, Dong-Cheng He, Guang-Zhou Tao, Yu-Suo Tong

**Affiliations:** 1 Department of Radiation Oncology, Huai’an First People’s Hospital, Nanjing Medical University, Huai’an, Jiangsu, China; 2 Cancer center, The Affiliated Hospital of Hang Zhou Normal University, Hangzhou, Zhejiang, China

## Abstract

The present study evaluated the clinical and prognostic value of neutrophil-to-lymphocyte ratio (NLR) in patients with locally advanced esophageal squamous cell carcinoma (ESCC) treated with definitive chemoradiotherapy (dCRT). A total of 517 patients with ESCC were enrolled and analysed retrospectively. The NLR was calculated at three time points: baseline, post-treatment, and at the time of tumor progression. Elevated NLR was defined as a ratio ≥5. High NLR at baseline was present in 204 (39%) patients and was significantly correlated with larger tumour size, advanced TNM stage, worse ECOG performance status, and dCRT response (*p* < 0.05). At a median follow-up of 17 months, patients with higher NLR at baseline had poorer progression-free survival (PFS) and overall survival (OS). On multivariate analysis, elevated NLR at baseline was independently associated with PFS and OS (HR = 1.529, *p* < 0.001 for PFS; HR = 1.856, *p* < 0.001 for OS). In addition, patients with high pre- and post-treatment NLR demonstrated worse clinical outcomes than other groups. Our results suggest that NLR is an independent prognostic indicator for patients with ESCC undergoing dCRT and changes in NLR level with treatment may indicate therapeutic benefit.

The prognosis of esophageal squamous cell carcinoma (ESCC) remains extremely poor in spite of improvements in surgical techniques, with a 5-year survival rate ranging from 15% to 25%^1^. Most patients are diagnosed at locally advanced stages (T3N1), and the concurrent chemoradiotherapy (CCRT) with or without surgery are widely accepted alternatives for curative treatment of these patients^2^,^3^. The clinical outcomes with this treatment have been comparable to those achieved with surgery alone^4^. However, the treatment failure rate after dCRT is high; approximately 56% of patients do not achieve complete response (CR) to dCRT^5^. Improved survival is more often observed in patients who achieve primary CR to dCRT compared with patients showing an incomplete response^6^. In addition, ineffective therapy for a resistant tumor could potentially reduce the quality of life in these patients. Therefore, the determination of molecular markers that can predict patients who would benefit from dCRT has important clinical implications.

Recently, several studies have revealed that the presence of an ongoing systemic inflammatory response is associated with adverse outcomes in variety of solid organ malignancies, including ESCC^7^,^8^. Such response may lead to tumor invasion, progression, and metastasis through recruitment of T lymphocytes, chemokines, aberrant activation of cytokines, suppression of apoptosis, DNA damage, and subversion of the adaptive immune system^9^. There are a number of parameters that can be used to measure systemic inflammation response, such as cytokine levels, modified Glasgow Prognostic Score (mGPS, which combines C-reactive protein and albumin), platelet-to-lymphocyte ratio (PLR), lymphocyte-to-monocyte ratio (LMR), and neutrophil-to-lymphocyte ratio (NLR)^10^,^11^,^12^. Among these parameters, NLR is an easily calculated, reproducible, and inexpensive marker of systemic inflammation response, and has been widely investigated as a predictive or prognostic factor in advanced stages of various kinds of cancer including ESCCC, renal cell carcinoma, gastric cancer, and prostate cancer^13^,^14^,^15^,^16^. Recently, Yao *et al*.^17^ reported that pre-treatment NLR was an independent prognostic factor for patients with non-small cell lung cancer; and that patients with high pretreatment NLR were resistant to platinum-based chemotherapy, indicating a role of NLR in chemotherapy resistance. However, the prognostic value of NLR in locally advanced ESCC treated with dCRT has not been studied previously. We hypothesised that ESCC patients with elevated NLR would show resistance to dCRT and poor survival.

Therefore, the aim of the present study was to examine the association of pretreatment NLR with treatment response rate, progression-free survival (PFS), and overall survival (OS) in patients with locally advanced ESCC treated with dCRT. We also analysed the impact of change of NLR with treatment to investigate its role as a response indicator.

## Methods

Criteria for reporting recommendations for tumor markers in prognosis study (REMARK) were followed wherever possible.

### Study population

The study protocol was approved by the Institutional Review Board for human studies of Huai’an First People’s Hospital, Huai’an, China; and informed written consent was obtained from all subjects. The study was performed in accordance with the approved guidelines. Patients with locally advanced ESCC treated with dCRT at the Nanjing Medical University Huai’an First Hospital between January 2006 and May 2010 were identified and retrospectively analysed. The inclusion criteria were as follows: (a) histologically confirmed primary ESCC by available biopsy specimens; (b) previously untreated; (c) Karnofsky score ≥70; (d) age ≤75 years; (e) no other significant medical disease. Patients with any evidence of active infection or presence of a chronic inflammatory condition were ineligible. Patients with hematology disease were excluded. Tumor were staged according to the conventional tumor-node-metastasis (TNM) classification for esophageal carcinoma (UICC, 6th edition), and the pretreatment clinical staging was based on the results of barium swallow, esophagogastroduodenoscopy (EGD), neck, chest, or abdominal CT examination, and bone scan.

### Data collection and definitions

Clinicopathologic characteristics including age, gender, Eastern Cooperative Oncology Group performance status (ECOG PS), tumor length, tumor differentiation, smoking status, and TNM stage were extracted from patients medial records. The laboratory data collected included hemoglobin concentration, absolute WBC count, absolute neutrophil count, and absolute lymphocyte count. At diagnosis, data on serum carcinoembryonic antigen (CEA) and squamous cell carcinoma antigen (SCCA) levels were also collected. The NLR was defined by dividing the number of absolute neutrophil count by the number of absolute lymphocyte count. A NLR of 5 or greater was considered elevated in accordance with previous studies^18^,^19^,^20^, and the cut-off point of ≥5 provided the strongest prognostic significance in our preplanned analysis. The values of NLR were calculated at three time points: baseline (pretreatment), post-treatment (within three days after dCRT), and at the time of progression.

### Definitive chemoradiotherapy

#### Chemotherapy

For patients with adequate bone marrow, renal, and hepatic function, chemotherapy was performed with a PF-based regimen (cisplatin/fluorouracil). Chemotherapy started on day 1, concurrent with initiation of radiotherapy. Cisplatin (80 mg per square metre of body surface area) was administered intravenously on Day 1, and fluorouracil (1 g per square metre of body surface area) was administered as a continuous infusion from Day 1 to Day 4. Two courses of chemotherapy were administered at a 4-week interval during radiotherapy.

#### Radiotherapy

All patients were treated with external-beam radiation using 6 or 15 MV LINAC (Siemens ONCOR). The radiation treatment was delivered as three-dimensional conformal radiation therapy or intensity-modulated radiation treatment to ensure tumour coverage and spare adjacent normal organs. Information from EGD examination and CT scan was studied in detail before delineation of target tumour volume. A total radiation dose of 50–60 Gy (1.8–2.0 Gy/fraction, 5 days per week) was given to the target of the gross oesophageal mass and enlarged lymph nodes.

### Clinical response evaluation and follow-up

To evaluate the treatment response, esophagogram, EGD and CT scan were performed 4 weeks after completion of dCRT. The response to treatment was assessed basically according to the following criteria. CR was defined as complete regression of all assessable lesions; partial response (PR) was defined as more than 50% reduction in primary tumor size or more of the sum of the lesions and no progression of assessable lesions; stable disease (SD) was defined as a reduction of <50% or increase <25% in tumor size; progressive disease (PD) was defined as an increase ≥25% in primary tumor volume or appearance of new lesions. We divided these categories into two groups: the effective group consisted o CR and PR, the resistant group consisted of SD and PD.

Follow-up evaluation was performed every 3 months for the first year, every 6 months for an additional 2 years, and then at the end of each year to study end or until death. During the follow-up period, diagnostic examinations were performed when recurrence and/or metastasis was suspected. Follow-up data were obtained from patients’ medical records and/or telephone interviews.

### Statistical analysis

The primary endpoints were OS and PFS. OS was defined as the time from diagnosis to death (event), or last follow-up (censored), and PFS was calculated from the date of therapy initiation to the time of disease progression (event), or last date of follow up (censored).

Continuous data were expressed as the median (range), and categorical variables were reported as frequencies and percentages. Continuous data were analysed using Mann-Whitney *U* test or Kruskal-Wallis test. Categorical variables were compared using Fisher’s exact test or chi-square test. Survival curves were plotted with Kaplan–Meier method, and the differences were compared using a log-rank test. Univariate and multivariate Cox regression analyses were performed to evaluate potential prognostic factors for survival, and only variables that showed statistical significance in univariate analysis were subsequently entered into multivariate analysis. All statistical analyses were conducted using SPSS Statistics version 20.0 (IBM, Inc.). A two-sided *p* value less than 0.05 was considered statistically significant.

## Results

### Patient characteristics and treatment outcomes

A total of 517 patients met the inclusion criteria and were selected for this study. Most of the patients were male (n = 407, 79%), and the median age at diagnosis was 65 years (range, 36 to 74 years). There were 83 (16%) cases with stage II disease, 377 (73%) cases with stage III disease, and 57 (11%) cases with stage IV disease. Median tumour length was 4 cm (range, 2 to 12 cm) and 224 primary tumours (43%) were longer than 5 cm. Detailed patient characteristics at baseline are shown in Table 1.

All 517 patients underwent concurrent dCRT with two cycles of PF. After treatment, CR, PR, SD, and PD were observed in 88 (17%), 203 (39%), 211 (41%), and 15 patients (3%), respectively. After dCRT, 17 patients (3%) underwent esophagectomy and 160 patients (31%) received adjuvant chemotherapy. With a median follow-up of 17 months (range, 2 to 76 months), 431 (83%) of the 517 patients died. Of these, the cause of death was progression of recurrent disease in 396 (92%) patients, treatment-related esophagoaortic fistula in 3 (1%) patients, and other causes in the remaining 32 (7%) patients. The median PFS and OS for the whole cohort of patients were 12 months and17 months, respectively.

For all patients, the median values for baseline serum WBC count, neutrophil count, lymphocyte count, and NLR were 5.86 × 10^9^/L (range, 2.87 to 16.00), 4.06 × 10^9^/L (range, 1.39 to 12.80), 1.12 × 10^9^/L (range, 0.28 to 3.47), and 3.24 (range, 0.85 to 19.28), respectively.

### Correlation between baseline NLR and clinicopathologic characteristics

At baseline, 204 (39%) patients had a high baseline NLR ≥ 5 and 313 (61%) patients had NLR < 5. The relationships between clinicopathologic variables and pretreatment NLR are shown in Table 2.

High NLR at baseline was significantly associated with worse ECOG PS (*p* = 0.033), larger tumor size (*p* < 0.001), distant lymph node metastasis (*p* < 0.001), advanced TNM stage (*p* < 0.001), and low response rate to dCRT (*p* < 0.001, Table 2). However, age, gender, tumor location, tumor differentiation, smoking status, hemoglobin concentration, CEA level, and SCCA level were not significantly different between the two groups (*p* > 0.05, Table 2).

### Baseline NLR and response to dCRT

Tumour responses to dCRT for the 517 patients are shown in Table 3. The objective response rate was significantly lower in patients with baseline NLR ≥ 5 than in patients with NLR < 5 (33 *vs.* 72%, *p* < 0.001, Table 3), indicating that NLR might be a predictive factor for dCRT in ESCC before treatment. However, there was no significant difference in dCRT response between post-treatment NLR ≥ 5 or <5 (52 *vs* 58%, *p* = 0.256, Table 3). The sensitivity of a low baseline NLR for predicting dCRT response was 72% (224/313) and the specificity was 67% (137/204). Unexpectedly, no significant correlations were observed between dCRT response and clinicopathologic parameters such as age, gender, tumor length, tumor location, tumor differentiation, and radiotherapy dose (Supplementary Table S1, *P* > 0.05).

### Prognostic significance of baseline NLR and other parameters

**Figure 1:**
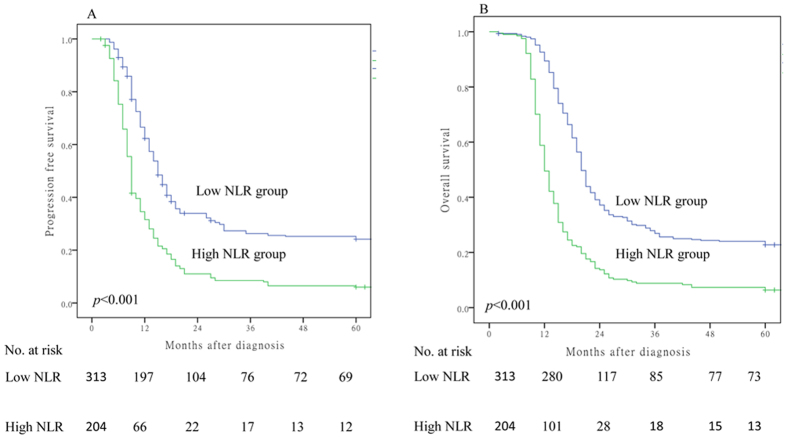
Association of baseline NLR (≥5 versus <5) with overall survival (**A**) and progression free survival (**B**).

To further examine whether pretreatment NLR was associated with outcomes of ESCC patients after dCRT, Kaplan–Meier survival analysis was used to compare the low (n = 313) and high (n = 204) NLR subgroups Fig. 1. Patients with high pretreatment NLR had worse PFS (NLR ≥ 5 *vs.* NLR < 5, median PFS 9 *vs.* 15 months, *p* < 0.001, Fig. 1A) and OS (NLR ≥ 5 *vs.* NLR < 5, median OS 12 *vs.* 20 months, *p* < 0.001, Fig. 1B).

Results of univariate analysis indicated that ECOG PS (≥2), tumor length (≥5), lymph node metastasis (positive), tumor stage (III/IV), dCRT response (SD + PD), SCCA level (≥1.5), and baseline NLR radio (≥5) were significantly correlated with decreased PFS or OS (*p* < 0.05, Table 4). All 8 clinicopathologic characteristics were therefore entered into subsequent multivariate analysis. The results of multivariate analysis revealed that pretreatment NLR, tumor stage, and dCRT response were independently prognostic factors of PFS and OS (Table 5).

### Changes in NLR and clinical outcomes

**Figure 2:**
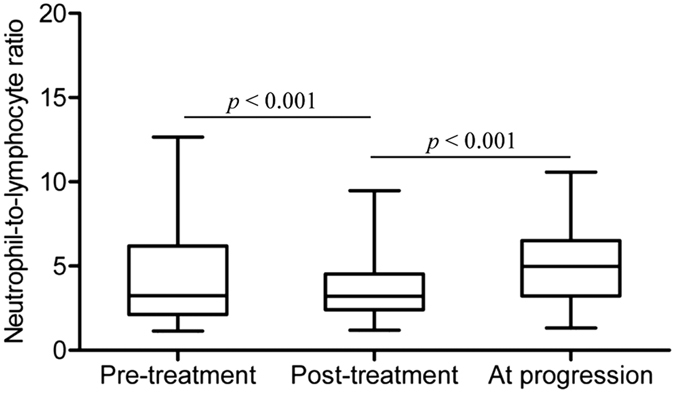
Change of NLR at baseline (pre-treatment, n = 517), after the completion of dCRT (post-treatment, n = 517), and at tumor progression (n = 436). Horizontal lines inside the box plots represent the median, boxes represent the interquartile range, and whiskers represent 97.5th and 2.5th percentiles.

We also examined changes in NLR values according to disease and treatment status. After dCRT, NLR decreased significantly (mean ± SD: baseline, 4.48 ± 3.05 *vs.* post-treatment, 3.87 ± 2.17, *p* < 0.001, Fig. 2) because of the effect of the treatment. However, NLR subsequently increased significantly to 5.04 ± 2.34 at tumour progression (p < 0.001 compared with the ratio after completion of dCRT, Fig. 2).

Patients were divided into four groups based on changes in NLR before and after dCRT (Table 6). (1) NLR ≥ 5 at baseline and after dCRT (n = 64, high-high group); (2) NLR ≥ 5 before dCRT and <5 after dCRT (n = 140, high-low group); (3) NLR < 5 at baseline and after dCRT (n = 251, low-low group); (4) NLR < 5 at baseline and ≥5 after dCRT (n = 62, low-high group). Patients in group 1 had significantly shorter PFS (median, 6 *vs.* 10 months, *p* < 0.001) and OS (median, 10 *vs.* 14 months, *p* < 0.001, Table 6) than those in group 2. However, patients in group 3 showed no significant differences from those in group 4 for PFS (median, 15 *vs.* 14 months, *p* = 0.720) and OS (median, 20 *vs.* 20.5 months, *p* = 0.793, Table 6).

## Discussion

The results of the present study supported our hypothesis and indicated that pretreatment NLR may be correlated with treatment response rate, PFS, and OS in patients with locally advanced ESCC treated with dCRT. In this retrospective study, patients with high pretreatment NLR (≥5) had a worse dCRT response rate and poorer PFS and OS. Although several studies have shown an association between NLR and prognosis of patients with ESCC, they mainly reported results for patients treated with surgery^21^,^22^. Moreover, our results also showed that patients with normalised post-treatment NLR (at 4 weeks after treatment) had a better PFS and OS than those with sustained high NLR. To our knowledge, this study is the first to assess clinical significance of NLR in patients with local advanced ESCC treated with dCRT.

As a biomarker of inflammation and immunology, increased NLR was previously correlated with advanced stage in endometrial cancer, small-cell lung cancer, and colorectal cancer^23^,^24^,^25^. Consistent with these reports, elevated NLR was also associated with advanced clinical stage and lymph node metastasis in the present study of ESCC. However, Sharaiha *et al*. examined a cohort of 295 esophageal cancer patients treated with esophagectomy and found no association between pretreatment NLR and tumor stage^8^. At present, it was difficult to explain such phenomena. The different pathological types could contribute to the different results.

Currently, definitive chemoradiotherapy with a PF regimen is an important component of the treatment of locally advanced ESCC, and the clinical CR to dCRT is widely accepted as the most important predictor of patient outcome^26^,^27^. However, chemoradiotherapy resistance and development of distant metastasis are major challenges in the management of ESCC^28^. Thus, in the present study, we focused markers related to systemic inflammation response that are known to be associated with chemotherapeutic efficacy. The role of NLR as a biomarker for evaluation of treatment response has been reported in several cancers treated with chemotherapy or radiotherapy, such as lung cancer, urothelial cancer, hepatocellular carcinoma, and prostate cancer^29^,^30^,^31^,^32^. In line with previous studies, a significant association between pretreatment NLR and dCRT response was observed in the current study. NLR was the only factor that showed a significant association with the dCRT response in ESCC. Consequently, the results of the present study provide important information to help physicians and patients make a more informed selection about the appropriateness of definitive chemoradiotherapy in ESCC.

Recently, mounting evidence indicates that the existence of systemic inflammation response, as evidenced by NLR, mGPS, CRP, and PLR, is correlated with a worse prognosis in patients with cancers^33^,^34^,^35^,^36^. The prognostic value of NLR has been demonstrated in many solid organ malignancies included in a recently published meta-analysis of 49 articles containing 14282 patients. These studies showed a broad prognostic impact of NLR across different cancer types, cancer stages, and treatments^9^. These findings have been replicated in the present study. In multivariate survival analysis, pretreatment NLR was an independent factor correlated with PFS and OS. The role of inflammation in carcinogenesis and tumor progression has been established during the past decade^37^, but the mechanisms connecting elevated NLR and poor outcomes remain elusive. Recent studies revealed that the presence of a systemic inflammation response could result in relative neutrophilia and lymphocytopenia. On one hand, neutrophils are able to secrete circulating vascular endothelial growth factors, which stimulate tumor angiogenesis, and release IL-1, IL-6, and TNF-alpha, which contribute to tumor progression^38^,^39^. Furthermore, relative neutrophilia could activate immunosuppression through inhibition of the activity of lymphocytes and other immune cells^40^. On the other hand, lymphocytes, usually CD4+ helper T and natural killers cells form the major component of the cell-mediated immune response to tumor infiltration and can attack tumor cells and eliminate nascent cancer cells^16^. A low lymphocyte count may be responsible for an inadequate host-to-tumor immunologic reaction with reduced response against tumor, leading to a worse clinical prognosis^25^,^41^. As NLR is calculated simply from the peripheral neutrophil count and lymphocyte count, a high NLR reflects enhanced neutrophil-dependent inflammatory response and/or a decreased lymphocyte-mediated anti tumor immune response, both of which contribute to tumor initiation, invasion, and metastasis. Baseline neutrophil and lymphocyte counts alone may provide limited reflection on the inflammatory response in tumor progression and are not independent prognostic factors for patients prognosis. Our findings indicated that the combination of neutrophil and lymphocyte provided more prognosis information than either component alone.

A previous study demonstrated the prognostic significance of pre- and post-treatment NLR in prostate cancer patients who received second-line chemotherapy^42^. The author observed that conversion from high to low NLR was associated with improved survival. In malignant mesothelioma, patients whose NLR normalised after 1 cycle of systemic therapy were found to have better prognosis compared with those whose NLR remained abnormal^43^. In our study, NLR values increased with tumor progression, and patients whose NLR remained ≥5 after dCRT had shorter PFS and OS than those whose NLR decreased to <5. These findings indicated that NLR might reflect the efficacy of treatment and help in monitoring progression of ESCC.

The present study provides the first clinical evidence supporting NLR as a biomarker for prognosis of patients with locally advanced ESCC. However, research limitations exist in our study. First, the retrospective design of the study may lead to bias, and the results must be validated in prospective study. Second, the total number of patients included is relatively small. In addition, unknown physiological factors that potential affecting the NLR might influence our results.

In conclusion, our results demonstrated that pretreatment NLR ≥ 5 was an independent prognostic factor for poor survival in patients with locally advanced ESCC treated with dCRT. Moreover, changes in NLR level with treatment may indicate therapeutic benefit.

## Additional Information

**How to cite this article:** Zhou, X.-L. *et al*. Neutrophil-to-lymphocyte ratio as a prognostic biomarker for patients with locally advanced esophageal squamous cell carcinoma treated with definitive chemoradiotherapy. *Sci. Rep.* **7**, 42581; doi: 10.1038/srep42581 (2017).

**Publisher's note:** Springer Nature remains neutral with regard to jurisdictional claims in published maps and institutional affiliations.

## Supplementary Material

Supplementary Information

## Figures and Tables

**Table 1:** Characteristics of patients.

Characteristics	Patients (%)
Age (y)	
Median	65
Range	36–74
Performance status (ECOG)	
0–1	355 (69%)
≥2	162 (31%)
Tumor length (cm)	
Median	4
Range	2–12
Location	
Proximal third	44 (9%)
Middle third	384 (74%)
Distal third	89 (17%)
Stage	
II	83 (16%)
III	377 (73%)
IV	57 (11%)
Radiotherapy dose (Gy)	
Median	50
Range	50–60
Chemotherapy cycle	
Median	2
Range	2–6

**Table 2:** Relationships between clinicopathological characteristics and pre-treatment NLR.

Characteristics	NLR radio		*p* value
	<5 (n = 313)	≥5 (n = 204)	
Age (years)			0.278
<60	47 (55.3)	38 (44.7)	
≥60	266 (61.6)	166 (38.4)	
Gender			0.584
Male	249 (61.2)	158 (38.8)	
Female	64 (58.2)	46 (41.8)	
Smoking at diagnosis			0.351
Never smoker	194 (59.0)	135 (41.0)	
Current or ex-smoker	119 (63.3)	69 (36.7)	
ECOG PS at diagnosis			0.033
0–1	226 (63.7)	129 (36.3)	
≥2	87 (53.7)	75 (46.3)	
Tumor location			0.237
Proximal third	31 (70.5)	13 (29.5)	
Middle third	225 (58.6)	159 (41.4)	
Distal third	57 (64.0)	32 (36.0)	
Tumor length (cm)			<0.001
<5	202 (68.9)	91 (31.1)	
≥5	111 (49.6)	113 (50.4)	
Tumor differentiation			0.892
Well	37 (57.8)	27 (42.2)	
Moderate	217 (61.0)	139 (39.0)	
Poor	59 (60.8)	38 (39.2)	
Node stage			0.158
N0	102 (56.4)	79 (43.6)	
N1	211 (62.8)	125 (37.2)	
Metastasis stage			<0.001
M0	293 (63.7)	167 (36.3)	
M1-lym	20 (35.1)	37 (64.9)	
Tumor stage			<0.001
II	65 (78.3)	18 (21.7)	
III/IV	248 (57.1)	186 (42.9)	
Hemoglobin concentration (g/L)			0.255
<120	73 (56.2)	57 (43.8)	
≥120	240 (62.0)	147 (38.0)	
SCCA at diagnosis (ng/ml)			0.365
<1.5	142 (62.8)	84 (37.2)	
≥1.5	171 (58.8)	120 (41.2)	
CEA at diagnosis (ng/ml)			0.705
<5	207 (61.2)	131 (38.8)	
≥5	106 (59.2)	73 (40.8)	
Adjuvant chemotherapy			0.496
Yes	93 (29.7)	220 (70.3)	
No	67 (32.8)	137 (67.2)	
dCRT response			<0.001
CR + PR	224 (77.0)	67 (23.0)	
SD + PD	89 (39.4)	137 (60.6)	

Abbreviations: M1-lym: distant lymph node metastasis, dCRT: definitive chemoradiotherapy, CR: complete response, PR: partial response, SD: stable disease, PD: progressive disease.

**Table 3:** Relationship between NLR categories and response to definitive chemoradiotherapy.

NLR	Case	CR + PR (%)	SD + PD (%)	*p* value
Baseline NLR < 5	313	224 (72%)	89 (28%)	<0.001
Baseline NLR ≥ 5	204	67 (33%)	137 (67%)	
Post-treatment NLR < 5	391	226 (58%)	165 (42%)	0.256
Post-treatment NLR ≥ 5	126	65 (52%)	61 (48%)	

Abbreviations: CR: complete response, PR: partial response, SD: stable disease, PD: progressive disease.

**Table 4:** Univariate analysis of factors associated with progression free survival and overall survival.

Prognostic factor	Case	Progression free survival			Overall survival		
		HR	95% CI	*p* value	HR	95% CI	*p* value
Age (continuous)	517	0.990	0.977–1.004	0.165	0.991	0.978–1.004	0.173
Gender							
Male	407	1			1		
Female	110	0.916	0.719–1.166	0.476	1.005	0.796–1.270	0.964
Smoking at diagnosis							
Never smoker	329	1			1		
Current or ex-smoker	188	1.089	0.890–1.332	0.408	1.029	0.844–1.254	0.776
ECOG PS at diagnosis							
0–1	335	1					
≥2	162	1.215	0.988–1.494	0.066	1.281	1.045–1.571	0.017
Tumor location							
Proximal third	44	1			1		
Middle third	384	0.997	0.669–1.487	0.988	1.058	0.753–1.487	0.746
Distal third	89	1.029	0.793–1.336	0.828	1.074	0.723–1.597	0.723
Tumor length (cm)							
<5	293	1			1		
≥5	224	1.313	1.082–1.594	0.006	1.334	1.101–1.614	0.003
Tumor differentiation							
Well	64	1			1		
Moderate	356	1.136	0.841–1.535	0.406	1.157	0.858–1.562	0.339
Poor	97	1.195	0.839–1.701	0.324	1.141	0.801–1.624	0.465
Node stage							
N0	181	1			1		
N1	336	1.281	1.044–1.571	0.018	1.324	1.081–1.622	0.007
Metastasis stage							
M0	460	1			1		
M1-lym	57	1.976	1.470–2.656	<0.001	1.744	1.296–2.346	<0.001
Tumor stage							
II	83	1			1		
III	377	1.891	1.409–2.539	<0.001	1.997	1.489–2.679	<0.001
IV	57	3.317	2.241–4.909	<0.001	3.038	2.051–4.500	<0.001
SCCA at diagnosis (ng/ml)							
<1.5	226	1			1		
≥1.5	291	1.209	0.995–1.470	0.056	1.226	1.011–1.486	0.038
CEA at diagnosis (ng/ml)							
<5	338	1			1		
≥5	179	1.003	0.819–1.228	0.976	1.045	0.857–1.276	0.662
Radiotherapy dose (Gy)							
50	364	1			1		
>50	153	1.052	0.851–1.301	0.638	1.132	0.920–1.394	0.256
Adjuvant chemotherapy							
Yes	160	1			1		
No	357	1.027	0.836–1.263	0.797	1.051	0.856–1.291	0.634
dCRT response							
CR + PR	291	1			1		
SD + PD	226	2.216	1.822–2.695	<0.001	2.284	1.883–2.772	<0.001
Baseline NLR radio							
<5	313	1			1		
≥5	204	2.157	1.774–2.624	<0.001	2.408	1.983–2.924	<0.001
Post-treatment NLR radio							
<5	391	1			1		
≥5	126	1.099	0.879–1.373	0.409	1.130	0.907–1.409	0.276

Abbreviations:, HR: hazard ratio, CI: confidence interval, M1-lym: distant lymph node metastasis, CR: complete response, PR: partial response, SD: stable disease, PD: progressive disease, ^*^P log-rank test.

**Table 5:** Multivariate analysis of factors associated with progression free survival and overall survival.

Prognostic factors	Progression free survival			Overall survival		
	HR	95% CI	*P*-value	HR	95% CI	*P*-value
ECOG PS at diagnosis (0–1 vs ≥2)	1.189	0.965–1.464	0.104	1.269	1.034–1.558	0.023
Tumor length (<5 vs ≥5)	1.172	0.959–1.432	0.121	1.150	0.944–1.402	0.166
Node stage (N0 vs N1)	1.085	0.862–1.365	0.488	1.135	0.905–1.424	0.272
Metastasis stage (M0 vs M1-lym)	0.848	0.544–1.321	0.465	0.705	0.452–1.100	0.124
Tumor stage (II vs III + IV)	1.715	1.254–2.347	0.001	1.722	1.261–2.353	0.001
SCCA at diagnosis (<1.5 vs ≥1.5)	1.079	0.885–1.316	0.452	1.129	0.928–1.373	0.224
dCRT response (CR + PR vs SD + PD)	1.815	1.473–2.231	<0.001	1.847	1.506–2.265	<0.001
Baseline NLR radio (Low vs High)	1.529	1.311–2.025	<0.001	1.856	1.498–2.300	<0.001

Abbreviations: HR: hazard ratio, CI: confidence interval, CR: complete response, PR: partial response, SD: stable disease, PD: progressive disease.

**Table 6:** Change in NLR and benefit from dCRT.

Baseline	Post-treatment	Case	Progression free survival		Overall survival	
			Median (95% CI)	*P* value	Median (95% CI)	*P* value
High (NLR ≥ 5)	High (NLR ≥ 5)	64	6 (5.04–6.96)	<0.001	10 (9.36–10.64)	<0.001
High (NLR ≥ 5)	Low (NLR < 5)	140	10 (8.95–11.05)		14 (12.95–15.05)	
Low (NLR < 5)	Low (NLR < 5)	251	15 (13.56–16.43)	0.720	20 (19.03–20.97)	0.793
Low (NLR < 5)	High (NLR ≥ 5)	62	14 (11.36–18.64)		20.5 (16.91–23.09)	

Abbreviations: CI: confidence interval.
