# Supplementary material for: Neutrophil-to-lymphocyte ratio as a prognostic biomarker for patients with locally advanced esophageal squamous cell carcinoma treated with definitive chemoradiotherapy
Source: Sci Rep. 2017 Feb 14;7:42581. doi: 10.1038/srep42581 (PMC5307338; doi:10.1038/srep42581)
Supplement: Supplementary Information [file srep42581-s1.pdf]

Supplementary information

Neutrophil-to-lymphocyte ratio as a prognostic biomarker for patients with locally advanced esophageal squamous cell carcinoma treated with definitive chemoradiotherapy

Xi-Lei Zhou[1†], Yong-Qiang Li[2†], Wei-Guo Zhu[1], Chang-Hua Yu[1], Ya-Qi Song[1], Wan-Wei Wang[1], Dong-Cheng He[1], Guang-Zhou Tao[1], Yu-Suo Tong[1 *]

[†]Xi-Lei Zhou and [†]Yong-Qiang Li contributed equally to this work and should be considered as joint first authors

[*]Correspondence to: Yu-Suo Tong, Email: tongyusuo@163.com

[1]Department of Radiation Oncology, Huai'an First People's Hospital, Nanjing Medical University, Huai'an, Jiangsu, China

[2]Cancer center, The Affiliated Hospital of Hang Zhou Normal University, Hangzhou, Zhejiang, China

Xi-Lei Zhou: zhouxilei1226@163.com

Yong-Qiang Li: liyongqiang19701217@126.com

Wei-Guo Zhu: jshazwg@126.com

Chang-Hua Yu: yu884443@sina.com

Ya-Qi Song: songaqi@163.com

Wan-Wei Wang: wly1019@126.com

Dong-Cheng He : hdcsuda@163.com

Guang-Zhou Tao: taoguangzhou01@163.com

Yu-Suo Tong: tongyusuo@163.com

SUPPLEMENTARY TABLE

Table S1. Univariate analysis with potential variables predicting tumor response to definitive chemoradiotherapy.

Variables	Case	Effective (CR+PR)	Resistant (PD+SD)	OR	95% CI	*p* value
Age				0.953	0.597-1.522	0.840
≤60	85	47	38			
>60	432	244	188			
Gender				0.950	0.621-1.454	0.814
Male	407	228	179			
Female	110	63	47			
Smoking at diagnosis				1.100	0.767-1.578	0.604
Never smoker	329	188	141			
Current or ex-smoker	188	103	85			

ECOG PS at diagnosis				1.252	0.862-1.820	0.238
0-1	355	206	149			
≥ 2	162	85	77			
Tumor location				1.557	0.813-2.980	0.181
Proximal third	44	29	15			
Middle third + Distal third	473	262	211			
Tumor length (cm)				1.003	0.706-1.424	0.988
< 5	293	165	128			
≥ 5	224	126	98			
Tumor differentiation				0.929	0.549-1.572	0.783
Well	64	35	29			
Moderate + Poor	453	256	197			
Node stage				1.154	0.800-1.663	0.444

N0	181	106	75			
N1	336	185	151			
Metastasis stage				1.620	0.932-2.817	0.087
M0	460	265	195			
M1-lym	57	26	31			
Tumor stage				0.959	0.598-1.538	0.862
II	83	46	37			
III+ IV	434	245	189			
SCCA at diagnosis (ng/ml)				1.369	0.963-1.948	0.080
< 1.5	226	137	89			
≥ 1.5	291	154	137			
CEA at diagnosis (ng/ml)				1.139	0.791-1.640	0.484
< 5	338	194	144			

≥ 5	179	97	82			
Radiotherapy dose (Gy)				1.124	0.769-1.644	0.545
60	364	208	156			
> 60	153	83	70			
Baseline NLR radio				5.146	3.513-7.538	< 0.001
< 5	313	224	89			
≥ 5	204	67	137			

Abbreviations: OR: odds ratio, CI: confidence interval, M1-lym: distant lymph node metastasis,, CR: complete response, PR: partial response, SD: stable disease, PD: progressive disease.
